# T-cell surveillance of the human brain in health and multiple sclerosis

**DOI:** 10.1007/s00281-022-00926-8

**Published:** 2022-04-01

**Authors:** Joost Smolders, Marvin M. van Luijn, Cheng-Chih Hsiao, Jörg Hamann

**Affiliations:** 1 Neuroimmunology Research Group, Netherlands Institute for Neuroscience, Amsterdam, The Netherlands; 2 MS Center ErasMS, Departments of Neurology and Immunology, Erasmus Medical Center, Rotterdam, The Netherlands; 3 MS Center ErasMS, Department of Immunology, Erasmus Medical Center, Rotterdam, The Netherlands; 4 Department of Experimental Immunology, Amsterdam institute for Infection and Immunity, Amsterdam University Medical Centers, Amsterdam, The Netherlands

**Keywords:** CNS, cerebrospinal fluid, perivascular space, meninges, T_RM_ cells, multiple sclerosis

## Abstract

Circulating and tissue-resident T cells collaborate in the protection of tissues against harmful infections and malignant transformation but also can instigate autoimmune reactions. Similar roles for T cells in the brain have been less evident due to the compartmentized organization of the central nervous system (CNS). In recent years, beneficial as well as occasional, detrimental effects of T-cell-targeting drugs in people with early multiple sclerosis (MS) have increased interest in T cells patrolling the CNS. Next to studies focusing on T cells in the cerebrospinal fluid, phenotypic characteristics of T cells located in the perivascular space and the meninges as well as in the parenchyma in MS lesions have been reported. We here summarize the current knowledge about T cells infiltrating the healthy and MS brain and argue that understanding the dynamics of physiological CNS surveillance by T cells is likely to improve the understanding of pathological conditions, such as MS.

## Introduction

The human central nervous system (CNS), comprising the brain and the spinal cord as well as the retina and the optic nerve, is regarded an organ sensitive to tissue damage with limited regenerative capacity. Acquired damage to brain or spinal cord usually results in long-term disability with sometimes a progressive loss of function. This delicate status requires an elaborate control of putative deleterious processes, which could accumulate in the initiation or promotion of tissue damage. In the case of immune surveillance by T cells, a carefully controlled balance is needed between prevention of infections versus uncontrolled, deleterious inflammatory responses. Protective T-cell immunity is indispensable for healthy CNS function and development. Among others, the absence of lymphocytes in *Rag*-knockout mice correlates with an impaired CD4^+^ T-cell-dependent hippocampal neurogenesis, and lesser abundance of CNS-patrolling T cells results in reduced brain-derived neurotrophic factor levels, impaired cognitive functioning, enhanced anxiety-like behavior, and impaired stress responses (reviewed by Ellwardt *et al.*, in [1]).

Besides promoting normal brain function, T cells are also key in controlling pathological conditions. In inborn or (drug-induced) acquired immune deficiencies, a higher risk of various opportunistic CNS infections has been reported [2]. Most notably, reactivation of the JC virus (also known as human polyomavirus 2) can cause a lethal white matter (WM) disease of the CNS called progressive multifocal leukoencephalopathy (PML) [3]. Although these diseases have been associated with a range of immune-suppressive agents, risk has been shown particularly high in patients treated with the drug natalizumab. Natalizumab selectively impairs migration of CD49d (VLA-4)-positive lymphocytes to the CNS and the gut. While the cascade leading to PML has been proposed to start with peripheral reactivation in the kidneys, this view was challenged by several studies showing presence of JC virus in postmortem CNS of unaffected individuals [4]. Likewise JC virus, herpes simplex virus (HSV) genetic material has been reported in postmortem healthy CNS tissue [5]. Immune-compromised individuals have an increased risk of developing HSV encephalitis. These data suggest that impairment of local T-cell control within the CNS could be instrumental in the local reactivation of these viruses. In addition to viral infections, an immunocompromised state has been associated with a higher risk of developing malignancies. As an example, in human immunodeficiency virus (HIV)-positive patients, primary CNS lymphoma is a manifestation of acquired immunodeficiency syndrome (AIDS). Altogether, insufficient T-cell surveillance of the CNS makes individuals vulnerable to pathological conditions, such as infections and certain malignancies.

On the other hand, exaggerated inflammatory processes are also harmful to healthy brain function and maintenance. When drawing focus to T cells, perforin, granzyme A, and granzyme B are highly neurotoxic [6]. In bacterial infections of the CNS, massive cytokine release can provide additional damage to CNS tissue and is usually treated with steroids. The most prevalent inflammatory disorder of the CNS is multiple sclerosis (MS), which is characterized by multifocal infiltration of T cells in the brain parenchyma and meninges. These MS infiltrates are associated with demyelination and axonal loss.

The tightly controlled environment of the CNS has been coined an immune-privileged site based on seminal experiments showing that antigen presentation within the borders of the CNS results in delayed T-cell responses compared to intra-thecal re-exposure of an extra-thecal antigen [7]. This feature of the CNS is maintained by a unique composition of compartments, barriers, and resident cell types, which shape the quantitative and qualitative presence of lymphocytes. In this review, we discuss the phenotypes of T cells infiltrating the CNS and how these are shaped within different compartments of the CNS. We will discuss the association of these cells with different pathological characteristics and disease stages of MS. Ultimately, we will discuss challenges ahead to further improve knowledge regarding T-cell surveillance of the human CNS.

## General concepts in T-cell surveillance and memory formation

In the human body, T cells play a key role in the maintenance of immune tolerance and in the defense against infections and cancer. During T-cell development in the thymus, self-reactive thymocytes are negatively selected by MHC class I- and II-expressing thymic epithelial cells and dendritic cells (central tolerance) [8]. Self-reactive clones that are able to escape this selection and end up in the circulation are further controlled by both T-cell-intrinsic and -extrinsic mechanisms (peripheral tolerance) [9]. In order to generate a memory T-cell armory responsive to the plethora of antigens, naive CD4^+^ and CD8^+^ T cells need to be educated by mature dendritic cells in secondary lymphoid organs, which requires antigen-specific TCR–MHC interaction, co-stimulation, and cytokine-mediated skewing. Eventually, high numbers of memory T cells persist and reside in peripheral tissues [10], ready to proliferate and exert their effector functions (e.g., cytokine production and cytotoxicity) upon encounter of the same antigen. Circulating central memory T (T_CM_) cells are poised for entering lymphoid tissues by expressing homing markers CCR7 and CD62L, whereas effector memory T (T_EM_) cells lack both molecules and are highly capable of migrating into non-lymphoid tissues [11]. More terminally differentiated memory cells re-express CD45RA (T_EMRA_), upregulate CD57, and lose CD28 expression, a process that corresponds to increasing age and chronic infection such as cytomegalovirus (CMV). In addition, memory T cells expressing high levels of CX_3_CR1 were found to be more prevalent in the T_EMRA_ versus T_EM_ compartment, reside in lymph nodes and show enhanced cytotoxicity and CMV specificity [12, 13]. The circulation provides the most accessible pool of T cells to be studied in the human body. Of all phenotypes discussed above, the circulation comprises on average more CD4 compared to CD8 T cells, with a sizeable proportion of naïve T (T_N_) cells. In normally aging individuals, proportions of circulating CD8^+^, more than CD4^+^, T_N_ cells are known to decrease in numbers [14].

More recently, also other memory T-cell populations have been described, such as stem cell-like (T_SCM_), peripheral (T_PM_), and tissue-resident (T_RM_) cells. T_SCM_ cells are abundant within lymph nodes and show a naive-like (CCR7, CD45RA) and memory (CXCR3, IL-2Rβ, CD95) phenotype [15, 16], probably representing a minimally differentiated memory population in between the T_N_ and T_CM_ compartment. T_PM_ cells express intermediate levels of CX_3_CR1 and can also self-renew and become either T_CM_ (CX_3_CR1^-^) or T_EM_/T_EMRA_ (CXR3C1^high^) cells, depending on antigen exposure. This subset patrols non-lymphoid tissues and can migrate back to the lymph node and blood [17]. When the proper antigen is encountered in such tissues, memory T cells upregulate a distinct set of markers including CD20, CD69, and/or CD103 in order to reside and provide local protection against microbial intruders (T_RM_ cells).

Besides the presence of CCR7 and CX_3_CR1, also expression of other chemokine receptors determines the tissue homing routes of T cells. For example, CCR7 (ligands: CCL19/21) works together with CXCR4 (ligand: CXCL12) for the entry of T_N_ and T_CM_ cells into secondary lymphoid organs [18–20]. Within these organs, T cells that express CXCR5 (ligand: CXCL13) are attracted to follicular borders and germinal centers to interact with cognate B cells. CCR4 (ligands: CCL17/22) and CXCR3 (ligands: CXCL9/10/11) are mainly involved in the recruitment of memory T cells into non-inflamed and inflamed tissue sites, respectively. CCR6 (ligand: CCL20) has been particularly associated with T cells crossing epithelial barriers and attacking inflamed tissues [21]. In several tissues including the spleen, mucosa, skin, lung, and brain, CXCR6 (ligand: CXCL16) has been proposed to contribute to local T-cell (T_RM_) organization [22–25]. Similar observations were made for CCR8 (ligand: CCL1) and CCR10 (ligands: CCL27/28), which are associated with T_RM_-cell formation in the skin [26, 27].

Moreover, chemokine receptor expression patterns are exemplary for the effector function of memory T cells. This not only accounts for their cytotoxic potential (e.g., CX_3_CR1), but also for the different types of cytokines that are produced upon activation. CCR6 is classically expressed by IL-17-producing helper T (Th17) cells. Differential CXCR3 and CCR4 expression further subdivides this population into IL-17^high^ (CXCR3^-^CCR4^+^; Th17), IL-17^int^ (CXCR3^+^CCR4^+^; Th17 double-positive), and IL-17^low^ (CXCR3^+^CCR4^-/dim^; Th17.1) cells [28, 29]. Th17.1 cells not only express high levels of IFNγ and GM-CSF, but also show resistance to both glucocorticoids and apoptosis and possess cytotoxic features [29–31]. IL-22-producing T (Th22) cells can be discriminated from Th17 cells based on CCR10 expression [32]. Memory T cells lacking CCR6 and IL-17 can be categorized into Th1 (CXCR3^+^CCR4^-^; producing IFNγ) and Th2 (CXCR3^-^CCR4^+^; producing IL-4) cells. CCR8 is mainly found on IL-10-producing regulatory T (Treg) cells, but also on small fractions of Th2 cells [33–35]. CCR6, CXCR3, and CCR4 can also be utilized to distinguish Th-like Treg subsets in a similar way as described above [36].

Obviously, these phenotypic and associated functional traits of human T cells have been most extensively characterized in *ex vivo* and in *in vitro* experiments with circulating cell fractions and have been correlated with functional insights acquired from animal studies. The uniqueness of the human CNS, in combination with normal immune aging and the environmental exposure to (neurotropic) viruses and other antigens, is likely to shape the circulating T-cell repertoire involved in physiological CNS surveillance.

## T-cell surveillance of the healthy CNS

**Fig. 1:**
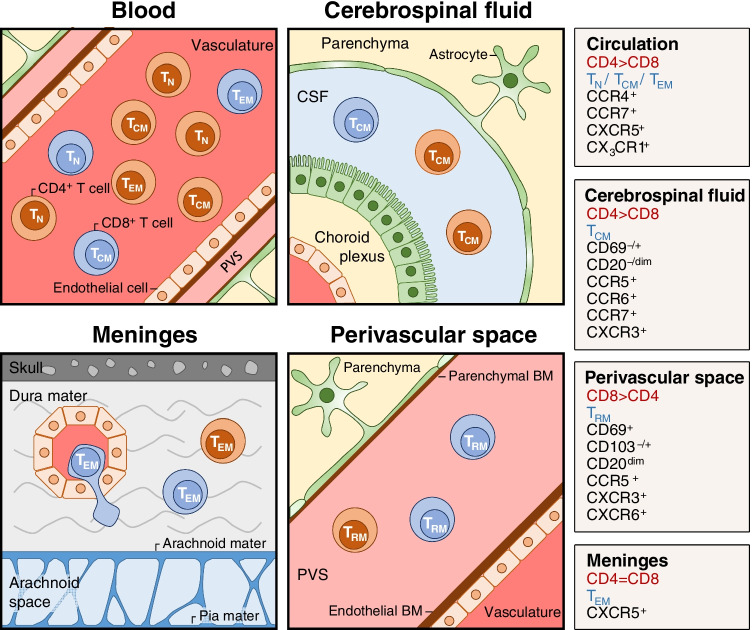
**Anatomical locations in the CNS relevant for T-cell surveillance.** Shown in clock-wise direction are the blood, the cerebrospinal fluid, the perivascular space, and the meninges with CD4^+^ (red) and CD8^+^ (blue) T cells. Boxes to the right provide details on the local T-cell CD4^+^ to CD8^+^ ratio, dominant phenotype, and surface marker profile. See the text for further details.  BM, basal membrane.

Immune surveillance of the CNS cannot be discussed separately from its anatomical organization (Figure 1). The strict compartmentation of the CNS plays a vital role in its tight control of infections and inflammatory responses. The CNS is engulfed by three layers of meninges, separating it from the periphery (reviewed by Papadopulos *et al*., in [37]). These layers surround the entire brain and spinal cord but also the optic nerve as an optical nerve sheet. The outer layer (dura mater) contains the venous sinuses, which drain blood from the cerebral veins. The inner meningeal layers of the arachnoid and pia mater are separated by the subarachnoid space containing cerebrospinal fluid (CSF). The superficial branches of the cerebrovasculature run through the perivascular space. The subarachnoid space is in a continuum with the perivascular space (PVS, see below), but also with the intracerebral ventricles containing the choroid plexus. The choroid plexus is a specialized epithelial organ and the main source of CSF. As such, a continuous stream of CSF circulates the perivascular space from the ventricles and is drained via the meningeal lymphatic structures and venous sinuses [38].

The pia mater overlies the brain parenchyma, which is lined with astrocyte endfeet-forming the glia limitans. This layer restricts and controls the movement of cells and molecules toward the parenchyma [39]. This layer adds to the tight control of inflammation within the brain parenchyma. Parenchymal cell populations show a limited capacity to start an inflammatory response. For instance, microglia are poor antigen presenters, and anti-inflammatory molecules as CD200 and CD47 are ubiquitously expressed and shed by multiple cell types [40]. The brain parenchyma is organized in grey matter and WM zones, with superficial cortical grey matter and deep grey matter areas containing neuronal cell bodies and WM containing axonal bundles. As such, cortical grey matter is for the largest part in close spatial contact with the subarachnoid space, while only a small part of subcortical WM is directly adjacent to ventricles.

The cerebrovasculature perfusing and draining the parenchyma comprises cells with specialized endothelium making up the blood–brain barrier (BBB) [41]. Tight junctions tightly control the movement of cells and large molecules. These blood vessels are surrounded by two basal membranes delineating the PVS. In contrast to the parenchyma, the PVS is an immunologically vibrant compartment. The PVS contains specialized macrophages and pericytes interacting with infiltrating immune cells. The PVS is again being covered by the astrocyte endfeet of the glia limitans. This whole structure of vessel, PVS, and glia limitans has been coined the neurovascular unit. Within the parenchyma, a stream of interstitial fluid (ISF) moves from efferent to afferent blood vessels (glymphatics), draining into the PVS and meningeal lymphatic structures. The CSF and ISF make up two independent compartments, draining via distinct routes [37].

CNS fluids, surfaces, and parenchyma host myeloid and lymphoid immune cells. How anatomical complexity as described above determines the localization and trafficking of immune cells and thereby shapes the immune responses in the CNS has been discussed in an excellent review by Ransohoff and Engelhardt [38]. The prime myeloid cells are phagocytic cells, comprising parenchymal microglia and non-parenchymal macrophages subsets in the choroid plexus, the perivascular space, and the meninges [42]. RNA sequencing of these cell populations has recently revealed unique transcriptional identities shaped by ontogeny and tissue environment [43]. In particular, microglia express genes setting them apart from other CNS macrophages. Brain-resident macrophages arise from embryonic yolk sac-derived erythromyeloid precursor (EMP) cells and self-renew during further development, while the contribution of bone marrow-derived monocytes is mostly restricted to choroid plexus macrophages [42]. In addition, recent work by Rustenhoven *et al.* showed that antigen-presenting cells populate the dural sinuses and can also interact here with patrolling lymphocytes [44]. In animal studies, tracers from CSF and ISF drain via lymphatic structures in the superficial and deep cervical lymph nodes [45, 46]. Although the exact number is uncertain, a frequently encountered estimation is that about 300 cervical lymph nodes are present in the human neck. Indeed, CSF-derived antigen-presenting cells were retrieved from the cervical lymph nodes of rodents, and stainings of human cervical lymph nodes showed presence of cells with phagocytosed CNS antigens [47, 48].

The most abundant lymphoid cells in the CNS are T cells. In fact, ≥90% of the cells in the CSF are T cells [49]. With 1,000–3,000 cells per ml, the total cell count in the CSF is 10–100-fold lower as compared to synovial or pleural fluid and 1,000–10,000-fold lower as compared to peripheral blood. Resident T_RM_ cells are found in the perivascular space and the meninges and at very low lower numbers in the parenchyma. Also here, they outnumber by far other lymphocytes, such as B and NK cells [50].

The first comprehensive characterization of T cells in CSF of patients without inflammatory diseases came from Kivisäkk et al [49, 51]. The ratio of CD4^+^ T cells to CD8^+^ T cells in the CSF is about 4 to 1. Most CSF CD4^+^ T cells display a CD45RA^−^CD45RO^+^CD27^+^CD62L^hi^CCR7^+^ phenotype similar to circulating CD4^+^ memory T cells. Notably, about 90% of CSF T cells express chemokine receptor CXCR3, which is expressed at higher levels compared to circulating memory T-cell fractions [51]. Subpopulations also express the α_4_β_7_ integrin, the chemokine receptors CCR4, CCR5, CCR6, and CCR9, and the activation and residency marker CD69 [51, 52]. Their phenotype suggests that these cells enter the CSF from the circulation to survey the subarachnoid space, where they can initiate immune responses and/or relocate to deep cervical lymph nodes. The accumulation of CCR6^+^ CD4^+^ T cells in CSF compared to blood, coinciding with expression of CXCR3, IFNγ, and GM-CSF suggests a Th17.1-like phenotype [31, 53]. These findings confirm the central role attributed to CCR6 in CNS homing by lymphocytes through the choroid plexus as revealed in experimental studies [54]. Recent single-cell RNA sequencing of T cells from CSF confirmed enrichment for memory CD4^+^ T cells with a CXCR3^+^KLRB1^+^ non-classical Th1 or Th17 phenotype as well as CD4^+^ and CD8^+^ T cells with a cytotoxic profile [50].

A site of T-cell accumulation passed by the CSF is the meninges in the dura [44]. Chemokines released from nearby stroma stimulate the extravasation of blood T cells circulating through the dural sinuses by triggering their CCR7, CXCR4, CXCR6, and CX_3_CR1 receptors. CD4^+^ and CD8^+^ T cells are found at a 1 to 1 ratio, and CD4^+^ T cells display characteristics of Th1, Th2, Th17, and Treg cells. The elegant work by Rustenhoven *et al*. points at a local interface where antigens brought by the CSF are captured by local antigen-presenting cells and are presented to patrolling T cells [44].

Other than the choroid plexus, releasing the CSF, and the dural sinuses, the BBB allows only minimal entry of circulating leukocytes into the cerebrovascular space, and almost none of these cells, under homeostatic condition, enter the parenchyma [55]. Examination of these resident T cells was initially limited to immunohistochemical approaches, which primarily revealed CD8^+^ granzyme B^−^perforin^−^ cells near the vasculature [56]. In 2013, we succeeded in isolating vital T cells from the corpus callosum of brain bank donors that came to autopsy within <10 h after death by a combination of mechanical and enzymatic dissociation, followed by Percoll gradient centrifugation [57]. We showed that these T cells were almost completely located in the perivascular space. Opposite to CSF, the ratio of CD4^+^ T cells to CD8^+^ T cells was about 1 to 2, and cells of both lineages had a late-differentiated CD45RA^−/low^CD27^−^CD28^−^ phenotype. They expressed CXCR3 and CX_3_CR1 that enable homing to inflamed endothelium and tissue, but hardly the lymph node-homing receptor CCR7. High expression of the IL-7 receptor α-chain CD127 suggested a role for IL-7 in their maintenance and absent/low production of perforin, granzyme A, and granzyme B a tight control of effector functions [57].

Evidence that T cells located in the brain may be bona fide T_RM_ cells was first obtained in mice, where CD8^+^ T cells persist independent from circulating memory T cells, provide protection against local vesicular stomatitis virus (VSV) infection, and partly express CD103 (α_E_β_7_) [58]. Increased numbers of CD69^+^CD103^-/+^ T cells were found after cerebral viral infections in human and mice [59]. More thorough investigation further clarified the phenotype of brain-resident human T cells [24]. CD103 presence on about half of all CD8^+^ T cells correlated with increased expression of the tissue-homing receptors CD49a (α_1_β_1_), CCR5, CXCR5, CXCR6, and CX_3_CR1, intermediate and low expression of the transcription factors T-bet and Eomes, increased expression of inhibitory molecules PD-1 and CTLA-4, and reduced expression of the cytolytic enzymes perforin and granzyme B. Despite being tightly controlled *in situ*, CD8^+^ T cells IFNγ, TNF, and GM-CSF—mostly in combination—upon stimulation *ex vivo*. CD4^+^ T cells also express CD69 but almost no CD103. Like their CD8^+^ counterparts, they express CD49a (α_1_β_1_), CCR5, CXCR5, CXCR3, CXCR6, CX_3_CR1, PD-1, and CTLA-4 [24]. A further property of CD8^+^ and CD4^+^ T cells is the production of granzyme K, enabling transendothelial diapedesis [60].

The phenotyping of T cells in the CNS disclosed major differences between cells in the CSF, the dural sinuses, and the cerebrovasculature with differences in, among others, CD4^+^ to CD8^+^ cell ratio, differentiation stage, migratory ability, and functional capacity. These differences seemingly exclude a direct exchange between the respective T-cell populations. There are actually also intriguing similarities, such as the increased percentage of T cells with an intermediate expression of CD20 that at incrementally higher levels is found in blood, CSF, and brain tissue of MS patients (see below) [61, 62].

## Role of T cells in the pathogenesis of multiple sclerosis

The most prevalent neuroinflammatory disease with a profound involvement of T cells in its disease process is MS. The outcomes from large genome-wide association studies and the modes of action together with the efficacy of existing immunotherapies, point toward circulating and CSF-homing CD4^+^ T cells (as well as B cells) as central players in the disease process of MS [63]. Naive CD4^+^ T cells from patients at risk of MS onset and progression contained separate sets of differentially expressed genes including *TOB1*, which was downregulated and is known to suppress T-cell proliferation [64, 65]. Genes involved in cell proliferation were also recently found in CSF CD4^+^ T cells as being discriminative for patients with newly diagnosed treatment-naive MS [66]. Furthermore, both *IL2RA* and *IL7RA* are associated with genetic risk variants for MS, and promote the development and skewing of CD4^+^ T cells expressing GM-CSF and IFNγ, respectively [67, 68].

In early MS patients, CCR6^+^ and not CCR6^-^ memory or naive CD4^+^ T cells were highly responsive to myelin peptides [69], suggesting that CCR6 expression demarks CD4^+^ memory T cells contributing to disease activity (reviewed by Van Langelaar *et al.*, in [70]). In humans, particularly GM-CSF^high^IFNγ^high^IL-17^low^ Th17.1 (CCR6^+^CXCR3^+^CCR4^-/dim^) cells are associated with MS-disease activity [29, 31, 71]. This pathogenic subset is selectively enriched in early MS and not in control CSF, expresses high VLA-4 levels and is preferentially targeted in clinical responders to natalizumab (anti-VLA-4 antibody). The CSF CD4^+^ T-cell pool contains more T_CM_ than T_EM_ cells during an acute relapse [72, 73], which suggests that T_EM_ cells are actively recruited from the CSF into the brain parenchyma. Within the human CCR6^+^ memory T-cell pool, Th17.1 cells reveal the highest IL-23 receptor and granzyme B expression and are most dominant in MS lesions. This may correspond to earlier findings in the experimental autoimmune encephalomyelitis (EAE) model that IL-23-skewed Th17 cells are cytotoxic for oligodendrocytes and neurons [74, 75]. Consecutive studies reported MS-associated memory CD4^+^ T cells with similar features as Th17.1 cells. B cells induce IFNγ-producing and both CCR6- and CXCR3-expressing ‘autoproliferative’ T cells in patients carrying the major HLA class II risk locus, which was attenuated by anti-CD20 therapy [76]. Using an unsupervised mass cytometry approach, Galli *et al*. demonstrated a CD4^+^ T-cell signature in MS blood that was classified by GM-CSF, IFNγ, and VLA-4 expression [77]. Herich *et al*. identified a VLA-4^high^ Th17.1-like subset expressing both CCR5 and granzyme K, which was highly capable of migrating across endothelial blood–brain layers *in vitro* [60]. Schafflick *et al*. found a cytotoxic CD4^+^ T-cell population to be enriched in MS CNS, also matching the Th17.1 cell [78]. Kaufmann *et al*. found a follicular T-cell subset that accumulated in the blood of natalizumab-treated MS patients and displayed high CD161 expression [79], another Th17.1-cell characteristic. The enhanced capacity of Th17.1 cells to cross endothelial blood–brain layers under non-inflamed *in vitro* conditions [29] was supported by a recent study [80]. The observation that CD4^+^ memory T-cell subsets are less able to pass tighter epithelial blood–CSF layers *in vitro* [80] could imply that preferential entry of Th17.1 cells via the BBB is an initial event in MS. Interestingly, in CD4^+^ T-cell-initiated EAE mice, myelin-reactive CD8^+^ T cells infiltrate the brain and not the spinal cord [81]. Although not proven yet, this supports a model for MS onset in which CD4^+^ T cells instigate BBB disruption and inflammation, making it possible for other immune subsets including B and CD8^+^ T cells to enter the brain and further mediate pathology.

Currently, it is relatively unclear whether peripheral CD8^+^ memory T cells are alternatively induced and promote or ameliorate the disease process of MS. On the one hand, EBV or CMV infection as a risk factor for MS contributes to functionally exhausted CD8^+^ T_EM/EMRA_ cells, through which for example B cells can escape from cytotoxicity-mediated killing [82, 83]. Distinct CD8^+^ T cells with regulatory functions have been found, including CXCR3^+^-, CD25^+^-, and HLA-E-restricted NKG2C^+^ subsets. On the other hand, CD8^+^ memory T cells expressing markers, such as CCR6, CD161, and CD20, were shown to be pathogenic in MS patients [61, 84]. Especially CD20^dim^ CD8^+^ (and CD4^+^) memory T cells may represent a pro-inflammatory, pre-T_RM_-like population, which is more abundant in the blood and CSF, expresses higher levels of brain-homing markers including VLA-4, CCR5, and CCR6, reveals increased myelin specificity, and is reduced by anti-CD20 antibody treatment in MS [61, 85]. Like CD4^+^ T cells, the outgrowth and effector functions of such CD8^+^ T cells are at least partially dependent on the presence of HLA-restricted protective (HLA-A*0201) and risk (HLA-A*0301) alleles [86, 87].

In addition to circulating and CSF T cells, phenotypic characteristics of T cells accumulating in meninges, perivascular space, and meninges have also been associated with the disease process of MS. The contribution of meningeal T cells to MS pathogenesis is likely to change during the course of MS. In early MS biopsies and autopsies, Lucchinetti *et al*. observed in 53 out of 138 donors cortical demyelination in numerical and spatial association with meningeal inflammatory infiltrates containing CD3^+^ T cells. Other studies also showed a positive correlation of these infiltrates with adjacent demyelination and axonal loss [88–90]. Meningeal inflammatory infiltrates contain, besides B cells, both CD4^+^ and CD8^+^ T cells [90–92]. Both a comparable proportion of CD4^+^ versus CD8^+^ meningeal T cells and a dominant presence of CD8^+^ meningeal T cells have been reported [92]. The precise phenotype of these T cells regarding markers of effector profile and tissue-residency is at present sparsely explored. The larger proportion of granzyme B-positive cells in MS meninges compared to controls suggests a larger proportion of cells with a cytotoxic potential [93].

Seraffini *et al*. showed that meninges of a subset of progressive MS donors contain lymphoid follicle-like infiltrates of B cells, T cells, and myeloid cells expressing surface markers of follicular dendritic cells and CXCL13 [94, 95]. These infiltrates were mostly found in the depths of cortical sulci and associated spatially with cortical subpial demyelination [96]. Furthermore, brain donors with follicles showed a greater extent of grey matter demyelination and a poorer prognosis in terms of earlier MS onset and reduced survival [95, 96]. The exact phenotype of T cells in these infiltrates is only partially disclosed. In accordance with the CXCL13 expression in these follicles, Bell *et al*. reported in the majority of follicles enrichment of CXCR5-positive follicular helper CD4^+^ T cells with increased expression of the activation marker CD69 [97]. However, meningeal cells were overall low in the expression of FoxP3 or PD-1. In cases with a high EBV viral load, Serafini *et al*. reported these follicles to contain large quantities of INFγ-positive CD8^+^ T cells [98]. Second, Magliozzi *et al*. and others found the presence of follicles to be associated with a history of relapses in progressive MS donors [97]. This suggests that infiltrating T-cell populations associated with relapses likely play a role in the origination of these structures.

The spatial association of meningeal T cells and follicles with cortical lesions, in combination with thorough experimental models, led to the hypothesis that soluble mediators produced by meningeal lymphocytes may accumulate in grey matter demyelination. Further characterization of cellular subsets within these structures may case more light on underlying mechanisms [96].

**Fig. 2:**
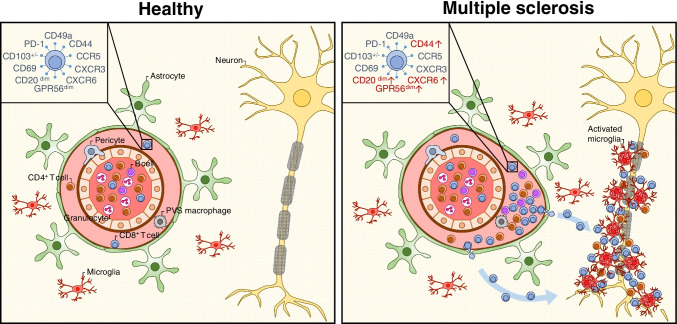
**Alterations in the neurovascular unit in multiple sclerosis.** In the healthy CNS, the PVS is populated by limited numbers of CD4^+^ (red) and CD8^+^ (blue) T_RM_ cells. In MS, T cells and fewer numbers of B cells (purple) accumulate in the PVS and enter the parenchyma, where T cells cluster together with myelin-collecting, foamy microglia near neuronal axons within demyelinating lesions. See the text for further details.

In MS normal-appearing WM (NAWM), more CD3^+^ T cells are encountered compared to WM of non-demented controls or Alzheimer’s disease [99, 100] (Figure 2). Likewise controls, NAWM T cells show a CD8^+^ over CD4^+^ dominance in MS [100, 101]. We could not find a correlation between the number of CD3^+^ T cells in the pyramid tract and axonal density. MS WM lesions as studied in autopsy material or diagnostic biopsies, are enriched for CD3^+^ T cells [102]. Frischer *et al.* and others showed that active and mixed active/inactive WM lesions showed a pronounced enrichment for CD3^+^ T cells, with inactive lesions showing comparable proportions to NAWM [99, 100]. Although CD8^+^ T cells are the most dominant fraction in both early and advanced disease, also numbers of CD4^+^ T cells are higher in these lesion types compared to NAWM [99–101, 103]. The presence of T cells in lesions correlated positively with histological markers of tissue damage and correlated negatively with age and disease duration [99, 103]. Most T cells in MS lesions and in NAWM are restricted to the PVS. Incidentally, perivascular cells form large cuffs of cells in de PVS, containing CD4^+^ and CD8^+^ T cells among other cells, such as B cells and myeloid cells. These cuffs likely reflect detrimental events, since their presence correlated with a progressive disease, a higher lesion load, and more mixed active/inactive lesions [100]. In analogy with meningeal follicle-like structures, Prineas proposed in the 1970s that these cuffs could be sites of local antigenic T-cell challenge [104]. In active and mixed active/inactive lesions, an increased proportion of CD4^+^ and CD8^+^ T cells infiltrate into the parenchyma [100, 105]. Whether these cells are engaging their specific antigens or are redistributed by a shift of local chemokine balance or otherwise changes in regulatory milieu due to local tissue damage and inflammation remains uncertain. Babbe *et al*. reported similar oligoclonal T-cell fractions to populate the PVS and the brain parenchyma, supporting no selective recruitment [106]. Van Nierop *et al*. found no significant increase in granzyme B production of parenchymal versus perivascular T cells, suggesting no specific antigen encounter [101]. Machado-Santos *et al*. postulated that perivascular T cells could contribute to tissue damage in MS by producing soluble inflammatory mediators in the perivascular space, likewise has been postulated for meningeal T cells [103].

Phenotypically, MS NAWM T cells show high similarity with control WM T cells. Van Nierop *et al*. described CD27^-^CD45RA^+/-^ CD8^+^ T cells to dominate MS NAWM and lesions matching our earlier general description of brain WM T cells [103]. CD8^+^ T cells isolated from MS NAWM and lesions and showed high expression of CD69 together with CD103, CD49a, PD-1, CD44, and CXCR6, which matched the phenotype of brain CD8^+^ T_RM_ cells from non-MS donors [100]. Accordingly, earlier immunohistochemical studies showed MS lesional and perivascular T cells to express CD69, CD103, CXCR3, and CCR5, and lack of CCR7 and S1P1 [72, 101, 103, 107]. Therefore, the majority of CD8^+^ T cells found in the MS brain displays a T_RM_-cell phenotype. MS CD4^+^ T cells have not been characterized to a similar extent. Despite the dominant T_RM_-cell phenotype, discrete MS-unique phenotypes of activated CD69^+^ T-cell clones could be hidden within the bulk of cells. Although we did not find differences in phenotypic profiles between MS and non-MS donors, other observed accumulation of small populations of CD4^+^ T cells within MS CNS infiltrates [79]. Interestingly, expression of scavenger receptor CD44 and chemokine receptor CXCR6 was increased in lesional versus control CD8^+^ brain T cells in our study. CD44 is a receptor for matrix proteins, which may affect the compartmentation of brain T_RM_ cells. On the other hand, CD44 is a receptor for osteopontin, which is highly expressed by lesional microglia and serves as a T-cell chemoattractant. CXCR6 is a receptor for CXCL16, which is also highly expressed by lesional myeloid cells. Additionally, we observed intermediate expression of CD20 to be enriched in CD8^+^ T cells from MS brain donors [62].

A small proportion of T cells in MS lesions showed expression of proliferation markers PCNA, Ki-67, nuclear expression of NFAT2, and early activation-markers CD137, indicating recent re-activation [100, 101, 103]. However, lesional cells may not be employing effector mechanisms. First, chronic activation of T cells can induce an exhausted state, resulting in a limited residual effector capacity [108]. Second, activation of CD8^+^ T cells can lead to local programmed cell death, resulting in selective maintenance of non-activated clones [103]. Accordingly, low proportions of cells positive for granzyme B have been found both in lesions and perivascular space [100, 101, 103]. Yet, lesional CD103^+^ CD8^+^ T cells showed higher expression of the pan-cytotoxicity marker GPR56. Lesional T cells were high in the expression of ICOS and TIM3 but not CD57, yet co-expression of these and other markers of T-cell exhaustion are unknown. Expression of anti-inflammatory TGF-β and IL-10 has not been shown on brain T cells [103]. Tzartos *et al*. reported immunoreactivity for IL-17 by CD4^+^ and CD8^+^ T cells, but also by oligodendrocytes and astrocytes in active and mixed active/inactive lesions [109]. Expression of IL-21 was observed in perivascular lymphocytes in MS regardless of the lesion type, although at a higher intensity in active and mixed active/inactive lesions [110]. Interestingly, IL-21 has been implicated in the prevention of CD8^+^ T-cell exhaustion in experimental chronic CNS infections and may hereby contribute to a sustained non-exhausted brain T_RM_-cell pool [111]. Whether and how other granzymes or cytokines contribute to the local function of brain T_RM_ cells remains to be determined.

## Upcoming challenges

Altogether, recent work contributed substantially to our understanding of the phenotypes of T cells in different CNS compartments. The striking difference in dominant T-cell phenotypes between circulation, CSF, meninges, and perivascular compartments highlights the need to study immune responses in local compartments. This phenotypic diversity could be dependent on several variables.

The migration of T cells from circulation to CSF and from CSF to parenchyma is a highly controlled process, with the choroid plexus, blood–brain barrier, and glia limitans being strictly organized barriers. These barriers require specific traits of crossing T cells, including expression of CXCR3, CCR6, P-selectin, and granzyme K to mediate migration from the circulation to the CSF or perivascular space [49, 54, 60]. The functional programs needed to enter the parenchyma are not fully understood, yet cytokines, as IL-17, have a limited effect on the glia limitans barrier [112]. Work in MS suggests the CXCR6 may contribute to the entry of T cells into the parenchyma in inflammatory lesions [100]. On the whole, the phenotypic profile of T cells is likely to adapt to functional properties acquired for tissue compartmentation.

Second, the interaction with other CNS-resident cell types is likely to provide co-stimulatory signals and soluble mediators, which affect functional programs in brain T cell. The subarachnoid space, PVS, meninges, and parenchyma are rich in specialized myeloid cells, which can provide not only the earlier mentioned signals, but also present antigens to infiltrating leukocyte populations [42]. As such, the interaction of CNS-infiltrating T cells with perivascular cells has been shown to be a critical feature of the neuroinflammatory response in EAE [113]. This interaction is not restricted to the borders of the CNS, since CNS myeloid cells can express CCR7, mediating migration of these fractions to secondary lymphoid organs and interacting with T cells there [47, 72].

Third, the recruitment of T cells into the CNS is likely to be antigen-dependent. Both CD4^+^ and CD8^+^ CSF memory T cells display an expansion of restricted clones in the absence of any neuroinflammatory disease [50], suggesting a controlled recruitment of these cells into the CNS. Since brain-resident T-cell populations arose in seminal experimental studies following neurotropic virus infections [58, 59], and an immune-compromised state is associated with a higher risk of neurotropic opportunistic infections, viruses or other pathogens with neurotropic potential encountered during life are likely candidates to drive the development of these T cells. In this perspective, the detection of JC and HSV genetic material in asymptomatic postmortem brain tissue could be very relevant [4, 5]. Although this may be an MS-specific feature, CNS local EBV-directed CD8^+^ T-cell responses have been shown [98]. The clonal overlap between perivascular space, meninges, and CSF remains, however, to be determined. EBV is of special interest, since it appears a perquisite to develop MS [114]. It is under debate to which extent EBV serves as antigen for CNS T cells in MS or rather potentiates the interaction between EBV-infected B cells and T cells as driver of oligoclonal T-cell recruitment into the CNS [70]. As such, other environmental risk factors, such as low exposure to vitamin D and adolescence obesity, and genetic risk factors for MS could have generic effects on composition and function of the CNS T-cell pool in people with and without MS [115–117]. Notably, since vitamin D also gains access into the CNS and is processed by resident cells into its anti-inflammatory active metabolite, local interaction with CNS T-cell activation can be postulated [118]. Beyond the possibility of viral antigens, preferential recruitment of mucosal-associated invariant T (MAIT) cells into the MS CSF and MS lesions has been reported, with also a higher expression of CCR5, CCR6, CXCR6 on these cells, compared to non-MAIT T cells [119, 120]. Since activation of MAIT cells is dependent on metabolites of riboflavin biosynthesis pathways in bacteria and yeasts, the presence of these cells suggests additional classes of antigens.

Understanding the dynamics of physiological CNS surveillance by T cells is likely to offer additional insights in the understanding of pathological conditions, such as MS. Current MS therapies can disrupt physiological immune surveillance and are of limited efficacy in advanced MS. Identification of critical steps in T-cell CNS recruitment, compartmentation, and maintenance in physiological and pathological conditions may provide novel targets for an effective but also safe treatment of MS.

## References

[1] Ellwardt E, Walsh JT, Kipnis J, Zipp F (2016). Understanding the Role of T Cells in CNS Homeostasis. Trends Immunol.

[2] Schmidt-Hieber M, Zweigner J, Uharek L (2009). Central nervous system infections in immunocompromised patients: update on diagnostics and therapy. Leuk Lymphoma.

[3] Cortese I, Reich DS, Nath A (2021). Progressive multifocal leukoencephalopathy and the spectrum of JC virus-related disease. Nat Rev Neurol.

[4] Wollebo HS, White MK, Gordon J (2015). Persistence and pathogenesis of the neurotropic polyomavirus JC. Ann Neurol.

[5] Marcocci ME, Napoletani G, Protto V (2020). Herpes Simplex Virus-1 in the Brain: The Dark Side of a Sneaky Infection. Trends Microbiol.

[6] Giuliani F, Goodyer CG, Antel JP (2003) Yong VW Vulnerability of human neurons to T cell mediated cytotoxicity. J Immunol 171(1):368–37. 10.4049/jimmunol.171.1.368 10.4049/jimmunol.171.1.368 12817020

[7] Medawar PB (1948). Immunity to homologous grafted skin; the fate of skin homografts transplanted to the brain, to subcutaneous tissue, and to the anterior chamber of the eye. Br J Exp Pathol.

[8] Klein L, Hinterberger M, Wirnsberger G, Kyewski B (2009). Antigen presentation in the thymus for positive selection and central tolerance induction. Nat Rev Immunol.

[9] Walker LSK, Abbas AK (2002). The enemy within: keeping self-reactive T cells at bay in the periphery. Nat Rev Immunol.

[10] Farber DL, Yudanin NA, Restifo NP (2014). Human memory T cells: generation, compartmentalization and homeostasis. Nat Rev Immunol.

[11] Sallusto F, Lenig D, Förster R (1999). Two subsets of memory T lymphocytes with distinct homing potentials and effector functions. Nature.

[12] Böttcher JP, Beyer M, Meissner F (2015). Functional classification of memory CD8(+) T cells by CX3CR1 expression. Nat Commun.

[13] Gordon CL, Lee LN, Swadling L (2018). Induction and Maintenance of CX3CR1-Intermediate Peripheral Memory CD8+ T Cells by Persistent Viruses and Vaccines. Cell Rep.

[14] Goronzy JJ, Weyand CM (2019). Mechanisms underlying T cell ageing. Nat Rev Immunol.

[15] Gattinoni L, Lugli E, Ji Y (2011). A human memory T cell subset with stem cell-like properties. Nat Med.

[16] Lugli E, Dominguez MH, Gattinoni L (2013). Superior T memory stem cell persistence supports long-lived T cell memory. J Clin Invest.

[17] Gerlach C, Moseman EA, Loughhead SM (2016). The Chemokine Receptor CX3CR1 Defines Three Antigen-Experienced CD8 T Cell Subsets with Distinct Roles in Immune Surveillance and Homeostasis. Immunity.

[18] Lee B, Sharron M, Montaner LJ (1999). Quantification of CD4, CCR5, and CXCR4 levels on lymphocyte subsets, dendritic cells, and differentially conditioned monocyte-derived macrophages. Proc Natl Acad Sci U S A.

[19] Phillips R, Ager A (2002) Activation of pertussis toxin-sensitive CXCL12 (SDF-1) receptors mediates transendothelial migration of T lymphocytes across lymph node high endothelial cells. Eur J Immunol 32:837–847.10.1002/1521-4141(200203)32:3%3C837::AID-IMMU837%3E3.0.CO;2-Q 10.1002/1521-4141(200203)32:3<837::AID-IMMU837>3.0.CO;2-Q 11870628

[20] Goedhart M, Gessel S, van der Voort R (2019). CXCR4, but not CXCR3, drives CD8+ T-cell entry into and migration through the murine bone marrow. Eur J Immunol.

[21] Meitei HT, Jadhav N, Lal G (2021). CCR6-CCL20 axis as a therapeutic target for autoimmune diseases. Autoimmun Rev.

[22] Günther C, Carballido-Perrig N, Kaesler S (2012). CXCL16 and CXCR6 are upregulated in psoriasis and mediate cutaneous recruitment of human CD8+ T cells. J Invest Dermatol.

[23] Kumar BV, Ma W, Miron M (2017). Human Tissue-Resident Memory T Cells Are Defined by Core Transcriptional and Functional Signatures in Lymphoid and Mucosal Sites. Cell Rep.

[24] Smolders J, Heutinck KM, Fransen NL (2018). Tissue-resident memory T cells populate the human brain. Nat Commun.

[25] Wein AN, McMaster SR, Takamura S (2019). CXCR6 regulates localization of tissue-resident memory CD8 T cells to the airways. J Exp Med.

[26] McCully ML, Ladell K, Andrews R (2018). CCR8 Expression Defines Tissue-Resident Memory T Cells in Human Skin. J Immunol.

[27] Kok L, Dijkgraaf FE, Urbanus J (2020). A committed tissue-resident memory T cell precursor within the circulating CD8+ effector T cell pool. J Exp Med.

[28] Paulissen SMJ, van Hamburg JP, Dankers W, Lubberts E (2015). The role and modulation of CCR6+ Th17 cell populations in rheumatoid arthritis. Cytokine.

[29] van Langelaar J, van der Vuurst de Vries RM, Janssen M (2018). T helper 17.1 cells associate with multiple sclerosis disease activity: perspectives for early intervention. Brain.

[30] Ramesh R, Kozhaya L, McKevitt K (2014). Pro-inflammatory human Th17 cells selectively express P-glycoprotein and are refractory to glucocorticoids. J Exp Med.

[31] Koetzier SC, van Langelaar J, Blok KM (2020). Brain-homing CD4+ T cells display glucocorticoid-resistant features in MS. Neurol Neuroimmunol Neuroinflamm.

[32] Duhen T, Geiger R, Jarrossay D (2009). Production of interleukin 22 but not interleukin 17 by a subset of human skin-homing memory T cells. Nat Immunol.

[33] Freeman MM, Ziegler HK (2005). Simultaneous Th1-type cytokine expression is a signature of peritoneal CD4+ lymphocytes responding to infection with Listeria monocytogenes. J Immunol.

[34] Soler D, Chapman TR, Poisson LR (2006). CCR8 expression identifies CD4 memory T cells enriched for FOXP3+ regulatory and Th2 effector lymphocytes. J Immunol.

[35] Barsheshet Y, Wildbaum G, Levy E (2017). CCR8+FOXp3+ Treg cells as master drivers of immune regulation. Proc Natl Acad Sci U S A.

[36] Halim L, Romano M, McGregor R (2017). An Atlas of Human Regulatory T Helper-like Cells Reveals Features of Th2-like Tregs that Support a Tumorigenic Environment. Cell Rep.

[37] Papadopoulos Z, Herz J, Kipnis J (2020). Meningeal Lymphatics: From Anatomy to Central Nervous System Immune Surveillance. J Immunol.

[38] Ransohoff RM, Engelhardt B (2012). The anatomical and cellular basis of immune surveillance in the central nervous system. Nat Rev Immunol.

[39] Quintana FJ (2017). Astrocytes to the rescue! Glia limitans astrocytic endfeet control CNS inflammation. J Clin Invest.

[40] Koning N, Bö L, Hoek RM, Huitinga I (2007) Down regulation of macrophage inhibitory molecules in multiple sclerosis lesions. Ann Neurol 62(5):504–14. 10.1002/ana.21220 10.1002/ana.21220 17879969

[41] Daneman R, Prat A (2015). The blood-brain barrier. Cold Spring Harb Perspect Biol.

[42] Prinz M, Erny D, Hagemeyer N (2017). Ontogeny and homeostasis of CNS myeloid cells. Nat Immunol.

[43] Van Hove H, Martens L, Scheyltjens I (2019). A single-cell atlas of mouse brain macrophages reveals unique transcriptional identities shaped by ontogeny and tissue environment. Nat Neurosci.

[44] Rustenhoven J, Drieu A, Mamuladze T (2021). Functional characterization of the dural sinuses as a neuroimmune interface. Cell.

[45] Louveau A, Smirnov I, Keyes TJ (2015). Structural and functional features of central nervous system lymphatic vessels. Nature.

[46] Aspelund A, Antila S, Proulx ST (2015). A dural lymphatic vascular system that drains brain interstitial fluid and macromolecules. J Exp Med.

[47] van Zwam M, Huizinga R, Melief M-J (2009). Brain antigens in functionally distinct antigen-presenting cell populations in cervical lymph nodes in MS and EAE. J Mol Med (Berl).

[48] van Zwam M, Huizinga R, Heijmans N (2009). Surgical excision of CNS-draining lymph nodes reduces relapse severity in chronic-relapsing experimental autoimmune encephalomyelitis. J Pathol.

[49] Kivisäkk P, Mahad DJ, Callahan MK (2003). Human cerebrospinal fluid central memory CD4+ T cells: evidence for trafficking through choroid plexus and meninges via P-selectin. Proc Natl Acad Sci U S A.

[50] Pappalardo JL, Zhang L, Pecsok MK et al (2020) Transcriptomic and clonal characterization of T cells in the human central nervous system. Sci Immunol 5:eabb8786. 10.1126/sciimmunol.abb8786 10.1126/sciimmunol.abb8786 PMC8567322 32948672

[51] Kivisäkk P, Trebst C, Liu Z (2002). T-cells in the cerebrospinal fluid express a similar repertoire of inflammatory chemokine receptors in the absence or presence of CNS inflammation: implications for CNS trafficking. Clin Exp Immunol.

[52] Kivisäkk P, Tucky B, Wei T (2006). Human cerebrospinal fluid contains CD4+ memory T cells expressing gut- or skin-specific trafficking determinants: relevance for immunotherapy. BMC Immunol.

[53] Restorick SM, Durant L, Kalra S (2017). CCR6+ Th cells in the cerebrospinal fluid of persons with multiple sclerosis are dominated by pathogenic non-classic Th1 cells and GM-CSF-only-secreting Th cells. Brain Behav Immun.

[54] Reboldi A, Coisne C, Baumjohann D (2009). C-C chemokine receptor 6-regulated entry of TH-17 cells into the CNS through the choroid plexus is required for the initiation of EAE. Nat Immunol.

[55] Mastorakos P, McGavern D (2019) The anatomy and immunology of vasculature in the central nervous system. Sci Immunol 4(eaav0492). 10.1126/sciimmunol.aav0492 10.1126/sciimmunol.aav0492 PMC6816468 31300479

[56] Loeffler C, Dietz K, Schleich A (2011). Immune surveillance of the normal human CNS takes place in dependence of the locoregional blood-brain barrier configuration and is mainly performed by CD3(+)/CD8(+) lymphocytes. Neuropathology.

[57] Smolders J, Remmerswaal EBM, Schuurman KG (2013). Characteristics of differentiated CD8(+) and CD4 (+) T cells present in the human brain. Acta Neuropathol.

[58] Wakim LM, Woodward-Davis A, Liu R (2012). The molecular signature of tissue resident memory CD8 T cells isolated from the brain. J Immunol.

[59] Prasad S, Lokensgard JR (2019). Brain-Resident T Cells Following Viral Infection. Viral Immunol.

[60] Herich S, Schneider-Hohendorf T, Rohlmann A (2019). Human CCR5high effector memory cells perform CNS parenchymal immune surveillance via GZMK-mediated transendothelial diapedesis. Brain.

[61] von Essen MR, Ammitzbøll C, Hansen RH (2019). Proinflammatory CD20+ T cells in the pathogenesis of multiple sclerosis. Brain.

[62] Hsiao C-C, Fransen NL, van den Bosch AMR (2021). White matter lesions in multiple sclerosis are enriched for CD20dim CD8+ tissue-resident memory T cells. Eur J Immunol.

[63] Farh KK-H, Marson A, Zhu J (2015). Genetic and epigenetic fine mapping of causal autoimmune disease variants. Nature.

[64] Corvol J-C, Pelletier D, Henry RG (2008). Abrogation of T cell quiescence characterizes patients at high risk for multiple sclerosis after the initial neurological event. Proc Natl Acad Sci U S A.

[65] Zastepa E, Fitz-Gerald L, Hallett M (2014). Naive CD4 T-cell activation identifies MS patients having rapid transition to progressive MS. Neurology.

[66] Hrastelj J, Andrews R, Loveless S et al (2021) CSF-resident CD4+ T-cells display a distinct gene expression profile with relevance to immune surveillance and multiple sclerosis. Brain Commun 3(fcab155). 10.1093/braincomms/fcab155 10.1093/braincomms/fcab155 PMC8574295 34761221

[67] Hartmann FJ, Khademi M, Aram J (2014). Multiple sclerosis-associated IL2RA polymorphism controls GM-CSF production in human TH cells. Nat Commun.

[68] Lee L-F, Axtell R, Tu GH et al (2011) IL-7 promotes T(H)1 development and serum IL-7 predicts clinical response to interferon-β in multiple sclerosis. Sci Transl Med 3:93ra68. 10.1126/scitranslmed.3002400 10.1126/scitranslmed.3002400 PMC3739690 21795588

[69] Cao Y, Goods BA, Raddassi K et al (2015) Functional inflammatory profiles distinguish myelin-reactive T cells from patients with multiple sclerosis. Sci Transl Med 7:287ra74. 10.1126/scitranslmed.aaa8038 10.1126/scitranslmed.aaa8038 PMC4497538 25972006

[70] van Langelaar J, Rijvers L, Smolders J, van Luijn MM (2020). B and T Cells Driving Multiple Sclerosis: Identity. Mechanisms and Potential Triggers. Front Immunol.

[71] Koetzier SC, Neuteboom RF, Wierenga-Wolf AF (2021). Effector T Helper Cells Are Selectively Controlled During Pregnancy and Related to a Postpartum Relapse in Multiple Sclerosis. Front Immunol.

[72] Kivisäkk P, Mahad DJ, Callahan MK (2004). Expression of CCR7 in multiple sclerosis: implications for CNS immunity. Ann Neurol.

[73] Schneider-Hohendorf T, Rossaint J, Mohan H (2014). VLA-4 blockade promotes differential routes into human CNS involving PSGL-1 rolling of T cells and MCAM-adhesion of TH17 cells. J Exp Med.

[74] Birkner K, Wasser B, Ruck T (2020). β1-Integrin- and KV1.3 channel-dependent signaling stimulates glutamate release from Th17 cells. J Clin Invest.

[75] Larochelle C, Wasser B, Jamann H (2021). Pro-inflammatory T helper 17 directly harms oligodendrocytes in neuroinflammation. Proc Natl Acad Sci U S A.

[76] Jelcic I, Al Nimer F, Wang J (2018). Memory B Cells Activate Brain-Homing, Autoreactive CD4+ T Cells in Multiple Sclerosis. Cell.

[77] Galli E, Hartmann FJ, Schreiner B (2019). GM-CSF and CXCR4 define a T helper cell signature in multiple sclerosis. Nat Med.

[78] Schafflick D, Xu CA, Hartlehnert M (2020). Integrated single cell analysis of blood and cerebrospinal fluid leukocytes in multiple sclerosis. Nat Commun.

[79] Kaufmann M, Evans H, Schaupp A-L (2021). Identifying CNS-colonizing T cells as potential therapeutic targets to prevent progression of multiple sclerosis. Med (N Y).

[80] Nishihara H, Soldati S, Mossu A (2020). Human CD4+ T cell subsets differ in their abilities to cross endothelial and epithelial brain barriers in vitro. Fluids Barriers CNS.

[81] Wagner CA, Roqué PJ, Mileur TR (2020). Myelin-specific CD8+ T cells exacerbate brain inflammation in CNS autoimmunity. J Clin Invest.

[82] Pender MP, Csurhes PA, Smith C (2014). Epstein-Barr virus-specific adoptive immunotherapy for progressive multiple sclerosis. Mult Scler.

[83] Pender MP, Csurhes PA, Burrows JM, Burrows SR (2017). Defective T-cell control of Epstein-Barr virus infection in multiple sclerosis. Clin Transl Immunology.

[84] Annibali V, Ristori G, Angelini DF (2011). CD161(high)CD8+T cells bear pathogenetic potential in multiple sclerosis. Brain.

[85] Sabatino JJ, Wilson MR, Calabresi PA (2019). Anti-CD20 therapy depletes activated myelin-specific CD8+ T cells in multiple sclerosis. Proc Natl Acad Sci U S A.

[86] Jilek S, Schluep M, Harari A (2012). HLA-B7-restricted EBV-specific CD8+ T cells are dysregulated in multiple sclerosis. J Immunol.

[87] Friese MA, Fugger L (2005). Autoreactive CD8+ T cells in multiple sclerosis: a new target for therapy?. Brain.

[88] Androdias G, Reynolds R, Chanal M (2010). Meningeal T cells associate with diffuse axonal loss in multiple sclerosis spinal cords. Ann Neurol.

[89] Lucchinetti CF, Popescu BFG, Bunyan RF (2011). Inflammatory cortical demyelination in early multiple sclerosis. N Engl J Med.

[90] Reali C, Magliozzi R, Roncaroli F (2020). B cell rich meningeal inflammation associates with increased spinal cord pathology in multiple sclerosis. Brain Pathol.

[91] Howell OW, Schulz-Trieglaff EK, Carassiti D (2015). Extensive grey matter pathology in the cerebellum in multiple sclerosis is linked to inflammation in the subarachnoid space. Neuropathol Appl Neurobiol.

[92] van Olst L, Rodriguez-Mogeda C, Picon C (2021). Meningeal inflammation in multiple sclerosis induces phenotypic changes in cortical microglia that differentially associate with neurodegeneration. Acta Neuropathol.

[93] Kooi E-J, Geurts JJG, van Horssen J (2009). Meningeal inflammation is not associated with cortical demyelination in chronic multiple sclerosis. J Neuropathol Exp Neurol.

[94] Serafini B, Rosicarelli B, Magliozzi R (2004). Detection of ectopic B-cell follicles with germinal centers in the meninges of patients with secondary progressive multiple sclerosis. Brain Pathol.

[95] Magliozzi R, Howell O, Vora A (2007). Meningeal B-cell follicles in secondary progressive multiple sclerosis associate with early onset of disease and severe cortical pathology. Brain.

[96] Howell OW, Reeves CA, Nicholas R (2011). Meningeal inflammation is widespread and linked to cortical pathology in multiple sclerosis. Brain.

[97] Bell L, Lenhart A, Rosenwald A (2019). Lymphoid Aggregates in the CNS of Progressive Multiple Sclerosis Patients Lack Regulatory T Cells. Front Immunol.

[98] Serafini B, Rosicarelli B, Franciotta D (2007). Dysregulated Epstein-Barr virus infection in the multiple sclerosis brain. J Exp Med.

[99] Frischer JM, Bramow S, Dal-Bianco A (2009). The relation between inflammation and neurodegeneration in multiple sclerosis brains. Brain.

[100] Fransen NL, Hsiao C-C, van der Poel M (2020). Tissue-resident memory T cells invade the brain parenchyma in multiple sclerosis white matter lesions. Brain.

[101] van Nierop GP, van Luijn MM, Michels SS (2017). Phenotypic and functional characterization of T cells in white matter lesions of multiple sclerosis patients. Acta Neuropathol.

[102] Lucchinetti C, Brück W, Parisi J et al (2000) Heterogeneity of multiple sclerosis lesions: implications for the pathogenesis of demyelination. Ann Neurol 47:707–717. 10.1002/1531-8249(200006)47:6%3C707::AID-ANA3%3E3.0.CO;2-Q 10.1002/1531-8249(200006)47:6<707::aid-ana3>3.0.co;2-q 10852536

[103] Machado-Santos J, Saji E, Tröscher AR (2018). The compartmentalized inflammatory response in the multiple sclerosis brain is composed of tissue-resident CD8+ T lymphocytes and B cells. Brain.

[104] Prineas JW (1979). Multiple sclerosis: presence of lymphatic capillaries and lymphoid tissue in the brain and spinal cord. Science.

[105] Barnett MH, Prineas JW (2004). Relapsing and remitting multiple sclerosis: pathology of the newly forming lesion. Ann Neurol.

[106] Babbe H, Roers A, Waisman A (2000). Clonal expansions of CD8(+) T cells dominate the T cell infiltrate in active multiple sclerosis lesions as shown by micromanipulation and single cell polymerase chain reaction. J Exp Med.

[107] Sørensen TL, Tani M, Jensen J (1999). Expression of specific chemokines and chemokine receptors in the central nervous system of multiple sclerosis patients. J Clin Invest.

[108] McLane LM, Abdel-Hakeem MS, Wherry EJ (2019). CD8 T Cell Exhaustion During Chronic Viral Infection and Cancer. Annu Rev Immunol.

[109] Tzartos JS, Friese MA, Craner MJ (2008). Interleukin-17 production in central nervous system-infiltrating T cells and glial cells is associated with active disease in multiple sclerosis. Am J Pathol.

[110] Tzartos JS, Craner MJ, Friese MA (2011). IL-21 and IL-21 receptor expression in lymphocytes and neurons in multiple sclerosis brain. Am J Pathol.

[111] Johnson LDS, Jameson SC (2009). Immunology. A chronic need for IL-21. Science.

[112] Horng S, Therattil A, Moyon S (2017). Astrocytic tight junctions control inflammatory CNS lesion pathogenesis. J Clin Invest.

[113] Bartholomäus I, Kawakami N, Odoardi F (2009). Effector T cell interactions with meningeal vascular structures in nascent autoimmune CNS lesions. Nature.

[114] Bjornevik K, Cortese M, Healy BC (2022). Longitudinal analysis reveals high prevalence of Epstein-Barr virus associated with multiple sclerosis. Science.

[115] Munger KL, Levin LI, Hollis BW (2006). Serum 25-hydroxyvitamin D levels and risk of multiple sclerosis. JAMA.

[116] Høglund RAA, Meyer HE, Stigum H (2021). Association of Body Mass Index in Adolescence and Young Adulthood and Long-term Risk of Multiple Sclerosis: A Population-Based Study. Neurology.

[117] International Multiple Sclerosis Genetics Consortium (2019) Multiple sclerosis genomic map implicates peripheral immune cells and microglia in susceptibility. Science 365(eaav7188). 10.1126/science.aav7188 10.1126/science.aav7188 PMC7241648 31604244

[118] Smolders J, Schuurman KG, van Strien ME (2013). Expression of vitamin D receptor and metabolizing enzymes in multiple sclerosis-affected brain tissue. J Neuropathol Exp Neurol.

[119] Willing A, Leach OA, Ufer F (2014). CD8^+^ MAIT cells infiltrate into the CNS and alterations in their blood frequencies correlate with IL-18 serum levels in multiple sclerosis. Eur J Immunol.

[120] Carnero Contentti E, Farez MF, Correale J (2019). Mucosal-Associated Invariant T Cell Features and TCR Repertoire Characteristics During the Course of Multiple Sclerosis. Front Immunol.

